# Early Identification of Childhood Asthma: The Role of Informatics in an Era of Electronic Health Records

**DOI:** 10.3389/fped.2019.00113

**Published:** 2019-04-02

**Authors:** Hee Yun Seol, Sunghwan Sohn, Hongfang Liu, Chung-Il Wi, Euijung Ryu, Miguel A. Park, Young J. Juhn

**Affiliations:** ^1^Department of Pediatric and Adolescent Medicine, Mayo Clinic, Rochester, MN, United States; ^2^Department of Health Sciences Research, Mayo Clinic, Rochester, MN, United States; ^3^Division of Allergic Diseases, Mayo Clinic, Rochester, MN, United States; ^4^Department of Pediatric and Adolescent Medicine and Internal Medicine, Mayo Clinic, Rochester, MN, United States

**Keywords:** early, identification, asthma, children, informatics, EHR

## Abstract

Emerging literature suggests that delayed identification of childhood asthma results in an increased risk of long-term and various morbidities compared to those with timely diagnosis and intervention, and yet this risk is still overlooked. Even when children and adolescents have a history of recurrent asthma-like symptoms and risk factors embedded in their medical records, this information is sometimes overlooked by clinicians at the point of care. Given the rapid adoption of electronic health record (EHR) systems, early identification of childhood asthma can be achieved utilizing (1) asthma ascertainment criteria leveraging relevant clinical information embedded in EHR and (2) innovative informatics approaches such as natural language processing (NLP) algorithms for asthma ascertainment criteria to enable such a strategy. In this review, we discuss literature relevant to this topic and introduce recently published informatics algorithms (criteria-based NLP) as a potential solution to address the current challenge of early identification of childhood asthma.

## Introduction

Asthma is the most common chronic illness of childhood, affecting up to 17% of children and representing one of most burdensome chronic diseases in the US (1–6). At present, there are no signs of declining trends in the prevalence of asthma among children and adolescents; rather, they continue to increase in many parts of the world (5–7). Furthermore, a delayed diagnosis of asthma, especially in young children is common (8–10). The emerging literature suggests that childhood asthma increases long-term morbidities (11), which could be mitigated by timely therapeutic interventions. This current awareness leads to relevant and consequential questions, (1) what are the various long-term morbidities of childhood asthma that can be reduced through timely identification and therapeutic intervention, (2) what is the magnitude of delay in identifying childhood asthma and why does it occur, (3) what can be done to reduce or eliminate delay in identification of childhood asthma, and (4) what is the role of informatics in clinical care and research for early identification of childhood asthma? Even though currently almost all hospitals and most office-based physicians have adopted electronic health records (EHR) (12, 13), there is yet to evolve a strategic approach for applying informatics tools to identify early childhood asthma. The authors address each of the questions above, specifically the emerging role of informatics in improving early identification of childhood asthma in the EHR era.

### Timely Identification and Intervention of Early Onset Asthma Might Reduce the Risk of Long-Term Morbidity

Early onset asthma is no longer just the domain of childhood but also affects long-term morbidity in adulthood. The Tasmanian study showed that children with early-onset asthma or wheezing episodes exhibited a lung function decline by the age of 7 years, compared to healthy children and this gap widened and persisted up to 45 years of age (11). Also, lower lung function at age 7 was associated with chronic obstructive pulmonary disease (COPD) and asthma-COPD overlap syndrome (ACOS). Long-term morbidity of childhood asthma has been corroborated by other studies (14, 15). The Tucson birth cohort study reported that transient wheezers during the first 6 years of life (which has been previously considered a non-pathological entity) demonstrated impaired lung function similar to those with persistent asthma and worse than late-onset wheezers at 16 years of age (16). Control of asthma through interventions (e.g., inhaled corticosteroid [ICS]) improved airway inflammation, respiratory symptoms, asthma exacerbations, rescue medication use, quality of life, and bronchial responsiveness, suggesting that early ICS intervention might have beneficial effects for those at risk of poor long-term asthma outcomes (17–19). In addition, the recent literature suggests that childhood asthma poses numerous health threats through asthma-associated infectious and inflammatory disease comorbidities (AIICs) (20–31). Both children and adults with asthma are at an increased risk of serious respiratory infections [e.g., pneumococcal pneumonia or invasive pneumococcal diseases (20–22), pertussis (23), and common upper respiratory infections (e.g., otitis media and strep infection) (24–26). Furthermore, AIICs are not limited to respiratory infections but also non-respiratory infections such as blood stream infection, appendicitis, herpes zoster (27–30) as well as inflammatory condition including Celiac disease (32). While these AIICs can cause a significant morbidity, the impact of AIICs in children with asthma is currently overlooked. Without a diagnosis of asthma, one has limited access to therapeutic interventions which may mitigate the risk of serious respiratory infections such as an AIIC (e.g., hospitalization-required severe pneumonia) (33). Thus, early identification of childhood asthma is a prerequisite step in the mitigation and prevention of poor long-term asthma outcomes (e.g., exacerbations, poorly controlled asthma and impaired lung function) and AIICs.

### A Delay in Identification of Asthma in Children and Adolescents Is Common and Why it Happens

A delay in identification of childhood asthma, especially in young children is still common (8–10). We previously reported that almost two thirds of children under 18 years of age had a delay in asthma diagnosis, and the delay was as long as 3 years after one met Predetermined Asthma Criteria (see **Table 2-1**) (8). Bisgaard et al. revealed that 32% of children, ages 1–5 years in the US and Europe (*n* = 9,490), reported recurrent respiratory symptoms (cough, wheezing, and shortness of breath), and 28% of children reported weekly asthma symptoms of whom only 20% had an asthma diagnosis and only 9.5% were receiving ICS (34). Also, the Lung Health Survey showed that 7.5 % of high school students with recurrent and significant asthma symptoms were not diagnosed with asthma (35). Consequently, a delayed asthma diagnosis was associated with increased urgent care visits suggesting suboptimal care and limited access to proper asthma therapy (10, 36, 37).

Among complex causes for delaying diagnosis of pediatric asthma, both conceptual and operational predicaments can be potentially addressed or improved in the EHR era. Conceptually, there is a lack of consensus on asthma diagnosis. Case in point, the NIH-led workshop discussed the core and supplementary predictors and outcome variables for asthma research (38), but left asthma ascertainment criteria undefined. Currently, only a few asthma ascertainment criteria based on medical record review exist and provide a basis for developing informatics tools for these asthma criteria enabling early detection of childhood asthma as described in the next section. In 2015, the Canadian Thoracic Society and Canadian Pediatric Society criteria for asthma provided succinct and practical approaches to make a diagnosis of asthma for preschoolers (39), consisting of (1) existence of airflow obstruction (e.g., recurrent asthma-like symptoms or exacerbations), (2) reversibility of airflow obstruction (e.g., favorable bronchodilator response), and (3) no evidence for alternative diagnosis(Table 1). As an alternative asthma criteria, Predetermined Asthma Criteria (PAC) (Table 2-1) which is conceptually similar to the Canadian Thoracic Society criteria, consists of (1) airflow obstruction, (2) reversibility and variability of airflow obstruction (e.g., recurrent wheezing with respiratory symptoms), and (3) no evidence of alternative diagnosis. Its usefulness in detection of asthma has been proven in numerous asthma epidemiology (20, 23–31, 40–47). Also Asthma Predictive Index (API) (Table 2-2) which was developed to identify young children at risk of developing asthma can also be considered as feasibility of application of API to retrospective studies has been recently established (48, 49). API includes frequent wheezing in addition to either one major risk factor (parental history of asthma or eczema) or two of three minor risk factors (eosinophilia, wheezing without colds, and allergic rhinitis). Apart from the conceptual challenges, operational challenges also contribute to the delayed diagnosis of asthma. Even if asthma ascertainment criteria are in place, it is still challenging and extremely labor-intensive for a physician or other abstractor to comprehensively review, collect, and interpret all necessary information from structured (e.g., test results) and unstructured data (e.g., recurrent asthma symptoms or favorable bronchodilator responses written in clinicians' narrative free text) (50). Overcoming these challenges through informatics technologies provides a tremendous opportunity not only to enhance early identification of asthma but also to provide optimal asthma management through a clinical decision support system (CDSS) for clinicians and their care teams. Therefore, clinical informatics approaches, including recently developed natural language processing (NLP) algorithms for PAC and API, to be discussed in the next section, address both conceptual and operational barriers to early identification of asthma in children and adolescents for asthma care and research.

**Table 1:** Operational diagnostic criteria for asthma in children 1–5 years of age, a Canadian Thoracic Society and Canadian Pediatric Society.

**1. Documentation of airflow obstruction**	
Preferred	Documented wheezing and other signs of airflow obstruction by physician or trained health care practitioner
Alternative	Convincing parental report of wheezing or other symptoms of airflow obstruction
**2. Documentation of reversibility of airflow obstruction**	
Preferred	Documented improvement in signs of airflow obstruction to SABA ± oral corticosteroids by physician or trained health care practitioner
Alternative^†^	Convincing parental report of symptomatic response to a 3-month trial of a medium dose of ICS (with as-needed SABA)
Alternative^‡^	Convincing parental report of symptomatic response to SABA
**3. No clinical evidence of an alternative diagnosis**	

† In children with frequent symptoms and/or one or more exacerbation requiring rescue oral corticosteroids or a hospital admission;

‡ *In children with mild intermittent symptoms and exacerbations, the diagnosis is only suggested because the accuracy of parental report of a short-term response to inhaled short-acting β2-agonists (SABA) may be unreliable due to misperception and spontaneous improvement of another condition. Because this is a weaker alternative diagnostic method, confirmation by direct observation when symptomatic is preferred. ICS Inhaled corticosteroids*.

**Table 2:** Two asthma ascertainment criteria which were used for developing NLP algorithms.

**2-1. PREDETERMINED ASTHMA CRITERIA (PAC)**	
Patients were considered to have *definite* asthma if a physician had made a diagnosis of asthma and/or if each of the following three conditions were present, and they were considered to have *probable* asthma if only the first two conditions were present:	
History of cough with wheezing, and/or dyspnea, OR history of cough and/or dyspnea plus wheezing on examination (airflow obstruction), Substantial variability in symptoms from time to time or periods of weeks or more when symptoms were absent (reversibility and variability of airflow obstruction) Two or more of the following: Favorable clinical response to bronchodilator Nonsmoker (14 years or older) Sleep disturbance by nocturnal cough and wheeze History of hay fever or infantile eczema OR cough, dyspnea, and wheezing regularly on exposure to an antigen Blood eosinophilia higher than 300 μL Positive wheal and flare skin tests OR elevated serum IgE Pulmonary function tests showing one FEV_1_ or FVC < 70% predicted and another with at least 20% improvement to an FEV_1_ of higher than70% predicted OR methacholine challenge test showing 20% or greater decrease in FEV_1_ Nasal polyps	
Patients were excluded from our previous study if any of these conditions were present (no evidence of alternative diagnosis):	
Pulmonary function tests that showed FEV_1_ to be consistently below 50% predicted or diminished diffusion capacity Tracheobronchial foreign body at or about the incidence date Hypogammaglobulinemia (IgG <2.0 mg/mL) or other immunodeficiency disorder Wheezing occurring only in response to anesthesia or medications Bullous emphysema or pulmonary fibrosis on chest radiograph PiZZ alpha_1_-antitrypsin Cystic fibrosis Bronchopulmonary dysplasia Mild pectus excavatum with respiratory symptoms Paradoxical vocal cord motion Other major chest disease such as juvenile kyphoscoliosis or bronchiectasis	
*FVC forced vital capacity; FEV1, forced expiratory volume in 1 s*.	
**2-2.ASTHMA PREDICTIVE INDEX (API)**	
**Major Criteria**	**Minor Criteria**
Physician diagnosis of asthma for parents Physician diagnosis of eczema for patient	Physician diagnosis of allergic rhinitis for patient Wheezing apart from colds Eosinophilia (≥ 4%)

*Asthma is determined by frequent wheezing episodes (two or more) plus at least one of two major criteria or two of three minor criteria*.

### Informatics Approach for Early Identification of Asthma in an EHR Era: Natural Language Processing (NLP)

The amount of EHR data has grown exponentially which provides a tremendous opportunity to leverage EHR data for clinical research and practice. NLP, one of prominent informatics techniques, has been demonstrated to be a promising way to automate chart review enabling large-scale studies that require information embedded in clinical free text (50–53). Although NLP has been successfully applied in various clinical applications (54–56), it has been observed that NLP has been underutilized in EHR-based clinical research (57).

The criteria for asthma ascertainment are mostly based on clinical information such as a history of respiratory symptoms and relevant information which are largely embedded in EHR as free text. Due to the large volume of EHR (primarily free text information), manual chart review to ascertain patient asthma is very costly, time consuming, error-prone, and often impractical for point of care and population-based studies (50). The capability of an NLP algorithm to extract, process, and classify information in free text is a key feature in enabling early identification of asthma in the EHR era (58). However, asthma ascertainment utilizing informatics has not been fully explored. A research team applied a machine learning technique on EHR data (i.e., codes, drugs, and clinical text) in order to identify children with asthma (59). Their approach relies largely on physician-diagnosed asthma and does not take into account the patient's asthma symptoms that could precede the physician's asthma diagnosis. Thus, it is not suitable for early identification of asthma. Also, this approach is not able to provide physicians with evidence of the likelihood of asthma that would assist in their clinical decision making. In order to tackle these challenges and achieve a timely-diagnosis of asthma, we have developed NLP algorithms (both rule-based and machine learning algorithms) for two existing asthma ascertainment criteria, PAC and API as described in the following.

To develop and test NLP algorithms for asthma, the availability of asthma ascertainment criteria based on retrospective EHR data is prerequisite. To our knowledge, two criteria (PAC and API) for asthma ascertainment using existing health records are suitable for developing their corresponding NLP algorithms. Both PAC and API are capable of determining the index date of asthma when the criteria are fulfilled, which is an important feature for clinical and epidemiological studies requiring temporal discernment for asthma as an exposure or outcome.

**Figure 1:**
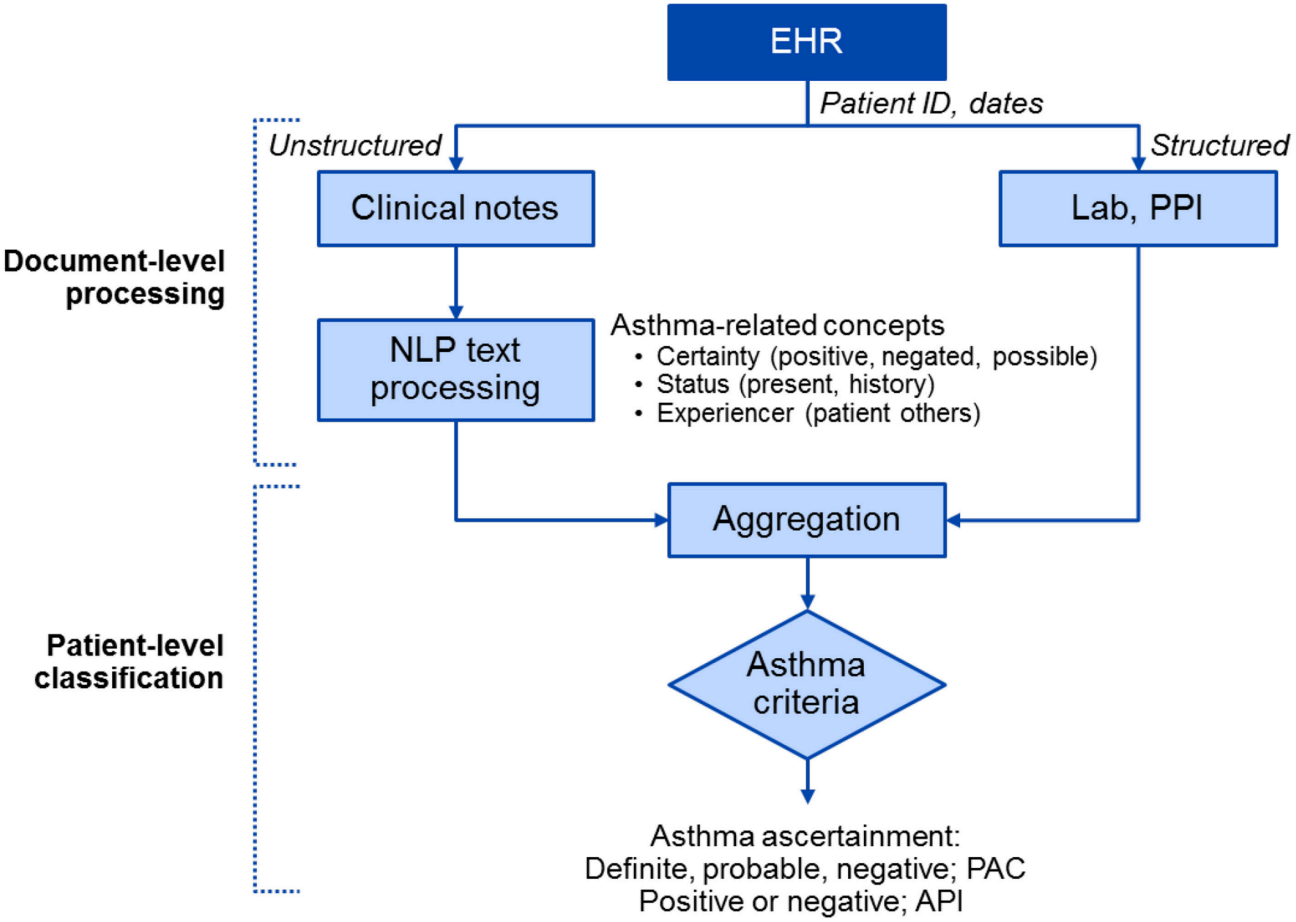
A high-level diagram of NLP algorithms for asthma ascertainment (i.e., NLP-PAC and NLP-API). There are two components in NLP algorithms: the document-level processing component extracts asthma-related concepts from unstructured data (clinical free text) using pattern-based rules and structured data (Lab and PPI) in asthma ascertainment criteria in Table 2, and the patient-level classification component aggregates processed information to ascertain asthma at a patient level. EHR, electronic health record; NLP, natural language processing; PPI, patient provided information; PAC, Predetermined Asthma Criteria; API, Asthma Predictive Index.

Conceptually, NLP algorithms extract, process and classify asthma-related events at the note level and then aggregate them to determine the patient asthma based on the given criteria (i.e., definite, probable, or negative of PAC; positive or negative of API) (Figure 1) (51, 60). Operationally, we applied an information extraction (IE)-based NLP pipeline for the development of NLP algorithms. For IE, we used various resource-driven tools which utilize domain-specific knowledge engineering such as MedTaggerIE for information in clinical documents (61) and MedTime for temporal information (62). Our original work on developing and testing an initial prototype NLP algorithm for PAC encompassed both rule-based and machine learning systems (50, 63).

We assessed performance of the prototype NLP algorithms for PAC in comparison with ICD code-based asthma ascertainment (50). The results of *the initial prototype* of our NLP algorithm for PAC demonstrated that NLP algorithms significantly outperformed ICD-code based asthma ascertainment in both validity and timely identification of asthma. Importantly, based on the asthma index date, defined as the earliest date of the constellation of symptoms meeting the PAC, 85% of children had experienced a delayed physician diagnosis by ICD code-based asthma ascertainment, compared to 27% by NLP algorithms. Subsequent to the testing of this initial prototype NLP we have been able to improve the performance. Our recently published study shows the performance of the NLP for PAC algorithm at 90% of PPV and 98% of NPV (51) which suggests a significant improvement over the prototype NLP algorithm. As this enables automated comprehensive chart review for the ascertainment of asthma almost real time and on a large scale, the commentary accompanying the paper considered this informatics work as “a giant step” for leveraging EHR for asthma care and research (64). This is especially true in the early identification of childhood asthma which allows timely intervention allowing mitigation of long-term and serious consequences of childhood asthma as discussed above.

Along these lines, we recently developed and tested an NLP algorithm for the API (60) using the manual chart review to ascertain asthma based on API as a reference. The performance of the NLP algorithm for API was 88% of PPV and 98% of NPV. Moreover, both NLP for PAC and API algorithms provide evidence (i.e., part of clinical text containing asthma-related events) for asthma ascertained by the algorithms, providing verifiable information for decision making of the clinicians. The promising results portend the potential implementation of these NLP algorithms for asthma care in a real-world setting as it greatly reduces the burden on clinicians in reviewing large volumes of EHR to discern asthma status. Currently, these two NLP algorithms are being further tested before implementation in our asthma care practice.

To ensure whether these NLP algorithms can be generalizable to other study settings with a different population, different clinical practice, and even different EHR system, we tested the performance of our current NLP algorithm for PAC in a different study setting with a different EHR system and demonstrated portability (PPV: 89% and NPV97% at a hospital in a different state) (65, 66). These results provide a new opportunity for large scale (multi-site) and automated identification of childhood asthma which may potentially address challenges associated with a delayed identification of asthma resulting in delayed therapeutic interventions and possible long-term morbidity.

### Implications of NLP Algorithms on Asthma Care and Research for Early Identification of Childhood Asthma

In addressing the existing challenges for early identification of childhood asthma as described above, NLP algorithms have potential impact for asthma care. *First*, incorporating our NLP algorithms for the two asthma ascertainment criteria as part of CDSS would support clinicians or their care teams according to their needs and workflow (e.g., alerting clinicians at a point of care or population surveillance). Also, those NLP algorithms provide retrieved events or evidence with temporal components supporting an asthma diagnosis, so clinicians can then review and discuss such evidence supporting the diagnosis of asthma with parents or caregivers. *Another important implication* of NLP algorithms for PAC and API is the ability for computational phenotyping subgroups of asthmatic children with distinctive clinical characteristics (i.e., stratification) based on the two independent asthma ascertainment criteria enabling precision medicine. For example, we recently reported that children who met both PAC and API criteria by NLP algorithms had clinical features characterizing children at high-risk for conditions such as atopy, poor asthma control, increased asthma exacerbation, impaired lung function, and high risk of asthma-associated infectious and inflammatory comorbid conditions (AIICs), compared to those who met only one criteria or healthy controls (32, 67). This computational phenotyping approach on a large scale is a novel way of identifying a high-risk population for either point-of-care or population surveillance. *Third*, along these lines, extending the application of NLP algorithms from early identification of asthma to the optimal management of asthma through *stratification and prognostication* of asthma is an important step to improve outcomes, care quality and ultimately care costs. Our group has developed and tested the multiple necessary NLP algorithms enabling optimal management of childhood asthma such as an NLP algorithm for asthma prognosis (e.g., likelihood of remission or relapse), triggers of asthma exacerbations (e.g., allergic sensitizations), and clinicians' adherence to guidelines (68–71). Currently, our group is testing the utility of an innovative asthma management strategy leveraging these NLP algorithms implemented in clinical work flow. *Lastly*, computational phenotyping through NLP algorithms is likely to significantly enhance clinical research capabilities for asthma. The algorithms have the potential to enable virtual clinical studies (e.g., clinical trial or observational studies) as they can automatically identify subjects who meet the enrollment criteria and determine outcomes collected prospectively or retrospectively. The conceptual feasibility of emulating clinical trials using EHR has already been demonstrated (72). This might truly revolutionize the current way of conducting clinical studies, especially randomized clinical trials and is an active research area under clinical research informatics.

## Summary

In this review, we summarized currently reported informatics approaches for the early identification of asthma and their potential to assist in timely identification so as to reduce the risk of long-term morbidity of asthma through improved management strategies. Given the current magnitude and impact of delayed identification of childhood asthma, we believe that the utilization of informatics approaches and techniques on EHR provides a great potential to improve clinical decision making in the early identification of asthma, allowing proper and timely diagnosis and management of asthma.

To enhance current asthma care and research through the use of rapidly emerging technologies to achieve early identification of asthma, multiple changes are necessary. *First*, awareness of the significance of early identification of childhood asthma is needed from its currently overlooked and misunderstood status, especially long-term impact of transient wheezing episodes and AIICs. *Second*, research effort for the development of new informatics tools, further tested and refined existing tools, and implementation of the clinical informatics tools such as NLP algorithms to the real world setting should be widely shared and supported. *Lastly*, not only the researchers but also clinicians who deliver direct individual patient care need to realize and adapt to the rapid advancement of informatics approaches leveraging EHR to enhance the current state of asthma care and research. Given the potential value and impact of informatics tools in the pursuit of delivering the best possible care to the patient, clinicians and researchers are strongly encouraged to seek a partnership with informatics teams to maximize the benefit of the patients they care for. The informatics approaches and tools for asthma discussed above can be implemented to enhance current asthma care and research, while they may help us make novel biomedical discoveries for asthma. We hope this review paper ignites such interest and endeavor.

## Author Contributions

HS and SS drafted the manuscript, HL, C-IW, ER, MP, and YJ provided critical input to the manuscript, and all the authors approved the final version.

### Conflict of Interest Statement

The authors declare that the research was conducted in the absence of any commercial or financial relationships that could be construed as a potential conflict of interest.
